# Dual-energy CT perfusion imaging for differentiating WHO subtypes of thymic epithelial tumors

**DOI:** 10.1038/s41598-020-62466-1

**Published:** 2020-03-26

**Authors:** Chunhai Yu, Ting Li, Ruiping Zhang, Xiaotang Yang, Zhao Yang, Lei Xin, Zhikai Zhao

**Affiliations:** 1 Imaging Department, Shanxi Tumor Hospital, The Affiliated Tumor Hospital of Shanxi Medical University, Taiyuan, Shanxi 030013 P.R. China; 2 Department of Nephrology, Taiyuan People’s Hospital, Taiyuan, Shanxi 030001 P.R. China

**Keywords:** Cancer imaging, Cancer imaging

## Abstract

To evaluate the role of conventional contrast-enhanced CT (CECT) imaging and dual-energy spectral CT (DECT) perfusion imaging in differentiating the WHO histological subtypes of thymic epithelial tumours (TETs). Eighty-eight patients with TETs who underwent DECT perfusion scans (n = 51) and conventional CT enhancement scans (n = 37) using a GE Discovery CT750 HD scanner were enrolled in this study. The mean maximal contrast-enhanced range (mean CEmax) and the perfusion and spectral parameters of the lesions were analysed. Among the six WHO subtypes (Type A, AB, B1, B2, and B3 thymoma and thymic carcinoma), the mean CEmax values and most of the perfusion and spectral parameter values of Type A and Type AB were significantly higher than those of the other subtypes (all P < 0.05), and there was no difference among Type B1, B2 and B3 (all P > 0.05). The mean CEmax value was not different between Type B (including Type B1, B2, and B3) and thymic carcinoma (P = 1.000). The PS, IC, NIC and λ_HU_ values in the optimal venous phase of thymic carcinoma were higher than those of Type B (all P < 0.05). The parameters of conventional CECT imaging and DECT perfusion imaging can help identify the subtype of TETs, especially those of DECT perfusion imaging in type B thymomas and thymic carcinomas.

## Introduction

Thymic epithelial tumours (TETs) account for approximately 20% of mediastinal tumours and 47% of anterior mediastinal tumours^1,2^. Pathological subtypes of TETs were determined by the World Health Organization (WHO) in 2004, including thymomas (Types A, AB, B1, B2 and B3) and thymic carcinoma (TC), based on the morphologic manifestations of the epithelial cells and the ratio of lymphocytes to epithelial cells^3^. In 2014, the International Thymic Malignancy Interest Group (ITMIG) confirmed the WHO histologic subtypes of TETs^4^. According to the different types of TETs, the clinical multidisciplinary team of the ITMIG adopted an individualized and appropriate treatment plan for each patient and predicted his or her clinical course and prognosis. Therefore, the noninvasive identification of TETs, and even of the pathological subtypes, is of clinical significance.

Preoperatively, different imaging modalities, including contrast-enhanced computed tomography (CECT), diffusion-weighted MRI (DWI), dynamic contrast-enhanced MRI (DCE-MRI), and 18-fluorine fluorodeoxyglucose positron emission tomography (FDG-PET), have been used to assess TETs^5–9^. According to the National Comprehensive Cancer Network (NCCN) guidelines for thymomas and thymic carcinomas in 2019, chest CT with contrast remains the first choice for imaging evaluation before treatment^10^. CECT can provide some general morphologic parameters (tumour size and shape, the presence of multiple nodules, calcification, capsule integrity, mean contrast-enhanced range [mean CEmax], etc.). However, there are many overlapping features among the histological subtypes of TETs, and certain difficulties in distinguishing different subtypes may be encountered^11,12^. Dual-energy CT (DECT) perfusion imaging can not only obtain the features found on conventional CT but also several quantitative and semiquantitative spectral parameters (water concentration, WC; iodine concentration, IC; the slope of spectral Hounsfield unit curve, λ_HU_) and perfusion parameters (blood flow, BF; blood volume, BV; mean transit time, MTT; permeability surface, PS) of tumours through a one-stop DECT perfusion scan. This CT scan method has been widely used in the evaluation of extramediastinal tumours, such as neck lymphoma and lung cancer^13–15^. Although only a few studies have used IC or perfusion parameters alone to evaluate TETs^16–19^, no studies have used the two different kinds of parameters to identify the different pathological subtypes of TETs simultaneously.

The six different TET subtypes have different biological characteristics and invasiveness and were divided into three subgroups according to increasing grade of malignancy—low-risk thymoma (LRT; Types A, AB and B1), high-risk thymoma (HRT; Types B2 and B3), and TC—in 2004^20^. Only one study^21^ has reported the comparison between low-risk (Types A and AB) and high-risk (Types B1, B2, B3 and TC) TETs using conventional enhancement CT. However, a large number of studies have compared LRT (Types A, AB, and B1), HRT (Type B2, B3), and TC^22–24^. Therefore, we aimed to regroup the six subtypes into three risk levels: LRT* (Types A and AB), HRT* (Types B1, B2 and B3), and TC. In this article, the subgroups were named Simplified Group 1 (LRT, HRT and TC) and Simplified Group 2 (LRT*, HRT* and TC) to facilitate the description of articles and data statistics.

In this retrospective study, we evaluated the role of quantitative and semiquantitative parameters of conventional CECT imaging and DECT perfusion imaging in differentiating the histological subtypes of TETs in the anterior mediastinum.

## Results

### General data

The clinical characteristics of the patients and pathological diagnoses of all TETs in the anterior mediastinum are listed in Table 1. The mean age of the 88 patients with TETs was 54.02 ± 9.93 years (range 31–78 years), in which the majority of patients with thymoma were female (37/57, 64.9%), while the majority of patients with TC were male (22/31, 71.0%) (*P* < 0.05). There was no significant difference in age among the six pathological subtypes of TETs (*P* = 0.064). According to the histological and immunohistochemical results, with regard to WHO pathological subtypes, there were 9 (10.2%) Type A, 8 (9.1%) Type AB, 13 (14.8%) Type B1, 16 (18.2%) Type B2, 11 (12.5%) Type B3, and 31 (35.2%) TC patients (Table 1).

**Table 1:** Clinical characteristics of 88 patients.

Clinical characteristics of 88 patients	WHO pathological subtypes						P
	Type A (n = 9)	Type AB (n = 8)	Type B1 (n = 13)	Type B2 (n = 16)	Type B3 (n = 11)	TC (n = 31)	
Sex							0.042
Male	3	4	5	5	3	22	
Female	6	4	8	11	8	9	
Age (mean year)	54.02 ± 9.93 (range 31–78 years)						
	59.89 ± 12.64	57.63 ± 10.56	49.23 ± 10.51	52.00 ± 7.68	47.36 ± 6.53	56.81 ± 11.16	0.064
Masaoka-Koga stage							<0.001
I	3	2	4	3	0	0	
II	5	5	6	5	2	0	
III	1	0	3	7	6	6	
IV	0	1	0	1	3	25	

TC, thymic carcinoma.

### Conventional CT features of 88 TETs

The distribution of conventional CT features among the six WHO subtypes is described in Table 2. The multiple nodules with fibrous septa (MNFS) and mean CEmax values were significantly different among the six subtypes of TETs (P = 0.002 and *P* < 0.001, respectively), while tumour size, ranging from 2.0 cm to 13.2 cm (mean diameter, 6.3 ± 2.4 cm) on the longest axis, was not significantly different based on the standard of 8 cm (*P* = 0.488). Calcification (25/88, 28.4%) could be found in each type, while MNFS (13/46, 28.3%) was only found in Types A, AB, B1 and B2.

**Table 2:** Relationship between some conventional CT features and WHO histologic subtypes in the 88 TETs.

Conventional CT features of 88 TETs	WHO pathological subtypes						*P*
	Type A (n = 9)	Type AB (n = 8)	Type B1 (n = 13)	Type B2 (n = 16)	Type B3 (n = 11)	TC (n = 31)	
Size							0.488
≥8 cm	4	3	2	5	5	7	
<8 cm	5	5	11	11	6	24	
Calcification	4	1	3	7	4	6	0.330
MNFS	4	3	4	2	0	0	0.002
Mean CEmax(HU)	63.89 ± 25.01 (n = 5)	45.13 ± 6.27 (n = 3)	30.31 ± 10.95 (n = 6)	29.56 ± 10.79 (n = 6)	26.55 ± 9.73 (n = 3)	28.39 ± 8.62 (n = 14)	<0.001

MNFS, multiple nodule with fibrous septum.

CEmax, the maximal contrast-enhanced range.

TC, thymic carcinoma.

### Mean CEmax values of the six WHO pathological subtypes and the two simplified groups of 37 TETs

Thirty-seven of 88 patients with conventional CECT scans were categorized into Type A (n = 5), Type AB (n = 3), Type B1 (n = 6), Type B2 (n = 6), Type B3 (n = 3) and TC (n = 14).

**Figure 1:**
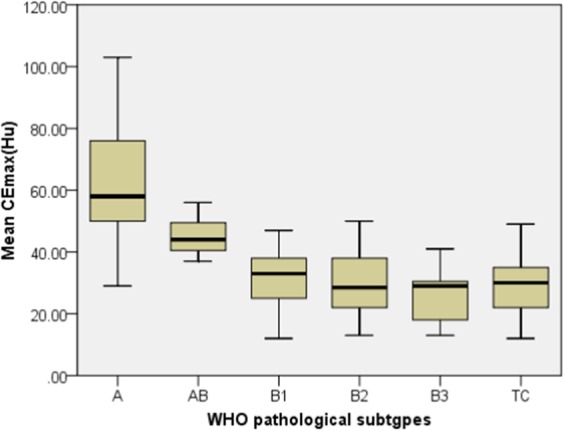
The relationship between mean CEmax(HU) and the WHO pathological subtypes of TETs. The mean CEmax values of type A or AB were significantly higher than for the other types (all *P* < 0.05), but there was no significant difference among Type B1, B2, B3, and TC (all *P* > 0.05).

The mean CEmax values of Type A (63.89 ± 25.01 HU) and Type AB (45.13 ± 6.27 HU) were significantly higher than those of the other subtypes (all *P* < 0.05), but there was no significant difference among Type B1, B2, B3, and TC (all *P* > 0.05) (Table 2, Fig. 1).

**Figure 2:**
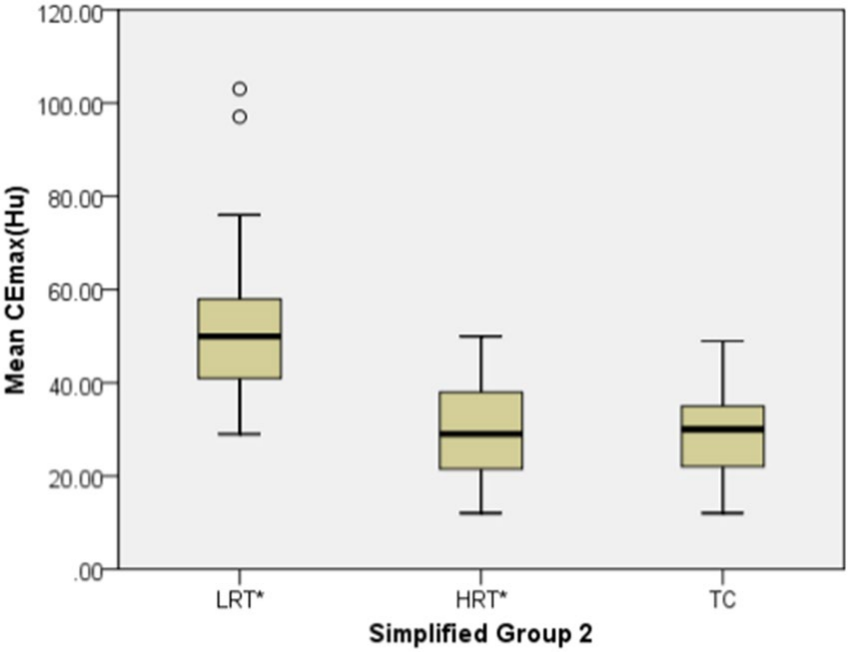
The relationship between mean CEmax (HU) and the Simplified Group 2 of TETs. The mean CEmax values of LRT* were significantly higher than that for HRT* and TC (all *P* < 0.001), but there was no significant difference between HRT* and TC (*P* = 1.000).

The mean CEmax values of LRT* (55.06 ± 20.57 HU) were significantly higher than those of HRT* (28.96 ± 10.41 HU) and TC (28.39 ± 8.62 HU) (all *P* < 0.001), but there was no significant difference between HRT* and TC (*P* = 1.000) (Table 3, Fig. 2).

**Table 3:** The mean CEmax values in Simplified Groups 1 and 2 of 37 TETs.

parameter	Simplified Group 1			*P*	Simplified Group 2			*P*
	LRT (A/AB/B1) (n = 14)	HRT (B2/B3) (n = 9)	TC (n = 14)		LRT* (A/AB) (n = 8)	HRT* (B1/B2/B3) (n = 15)	TC (n = 14)	
Mean CEmax(HU)	44.33 ± 16.36	28.33 ± 9.74	28.39 ± 8.62	<0.001	55.06 ± 20.57	28.96 ± 10.41	28.39 ± 8.62	<0.001

CEmax, the maximal contrast-enhanced range.

TC, thymic carcinoma.

### DECT perfusion imaging parameters of the WHO pathological subtypes of 51 TETs

**Figure 3:**
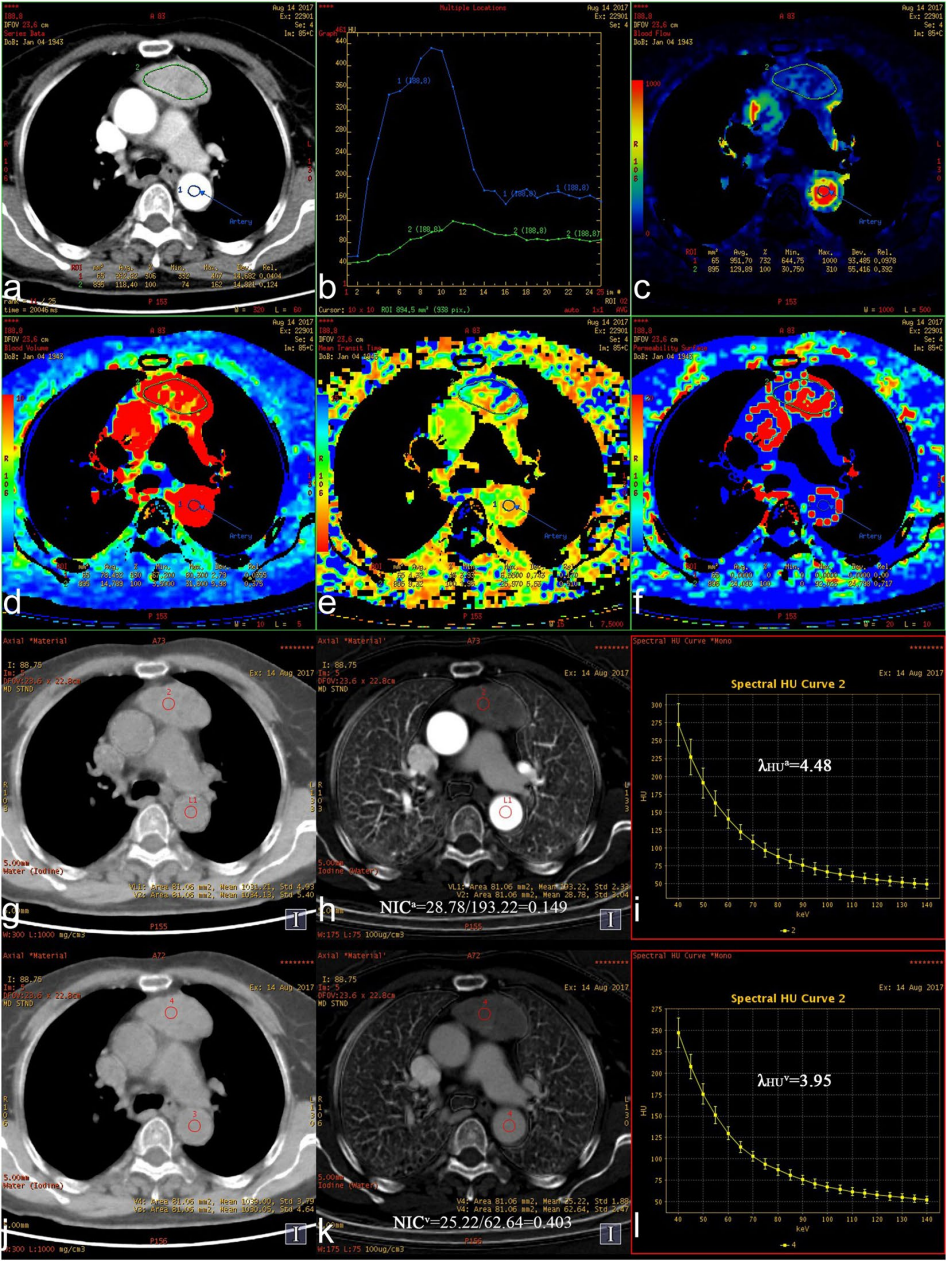
(**a–l**) Axial images showing a thymic carcinoma (75 y, M). **(a)** Original image (mixed energy) of the chest showing a left anterior mediastinal mass. ROI 1 was selected from the aorta to calculate the TDC curve automatically. ROI 2 was selected from the tumor to calculate perfusion parameters automatically. **(b)** The optimal arterial phase (tenth) and venous phase (twenty-first) were obtained in the TDC curve. (**c**,**d**,**e**,**f**) BF, BV, MTT, and PS value were 61.71 ml/min/100 g, 7.03 ml/100 g, 9.47 s and 41.33 ml/min/100 g respectively, in pseudo-color maps. (**g**,**h**, **i**) WC^a^, IC^a^, NIC^a^ and λ_HU_^a^ values of the tumor were 1038.60 mg/cm^3^, 11.83 × 10^2^ μg/cm^3^, 0.087, and 1.90 in the Water (Iodine), Iodine (Water) and spectral curve maps respectively, in the optimal arterial phase. (**j**,**k**,**l**) WC^v^, IC^v^, NIC^v^ and λ_HU_^v^ values of the tumor were 1040.87 mg/cm^3^, 14.18 × 10^2^ μg/cm^3^, 0.357, and 2.28 in the Water (Iodine), Iodine (Water) and spectral curve maps respectively, in the optimal venous phase.

Fifty-one of 88 patients with DECT perfusion scans were categorized into Type A (n = 4), Type AB (n = 5), Type B1 (n = 7), Type B2 (n = 10), Type B3 (n = 8) and TC (n = 17). For all DECT parameters, there were significant differences between the six subtypes, except for WC^a^, WC^v^ and MTT (Table 4, Fig. 3).

**Table 4:** Perfusion and spectral parameters in the different WHO pathological subtypes of 51 TETs.

Perfusion, Spectral parameters	WHO pathological subtypes						
	Type A (n = 4)	Type AB (n = 5)	Type B1 (n = 7)	Type B2 (n = 10)	Type B3 (n = 8)	TC (n = 17)	*P*
BF(ml/min/100 g)	169.66 ± 18.08	145.38 ± 12.37	57.94 ± 20.30	71.47 ± 20.49	45.46 ± 8.34	53.62 ± 13.84	<0.001
BV(ml/100 g)	26.60 ± 11.37	15.98 ± 4.16	6.74 ± 3.02	6.61 ± 1.76	5.16 ± 0.92	6.21 ± 1.89	<0.001
MTT(s)	11.27 ± 3.88	8.51 ± 1.71	8.90 ± 1.40	7.27 ± 1.40	9.88 ± 2.45	9.86 ± 4.07	0.134
PS(ml/min/100 g)	36.79 ± 10.31	35.06 ± 7.76	15.15 ± 7.67	23.28 ± 10.99	21.40 ± 7.11	28.92 ± 11.68	0.004
WC^a^(mg/cm^3^)	1038.62 ± 4.03	1032.95 ± 0.95	1040.18 ± 1.38	1034.47 ± 6.13	1032.86 ± 3.20	1034.78 ± 5.55	0.070
IC^a^ (×10^2^ μg/cm^3^)	38.45 ± 5.34	29.38 ± 1.43	9.01 ± 0.59	6.60 ± 2.23	8.85 ± 2.33	12.35 ± 4.94	<0.001
NIC^a^	0.244 ± 0.034	0.157 ± 0.006	0.083 ± 0.006	0.060 ± 0.021	0.063 ± 0.011	0.090 ± 0.029	<0.001
λ_HU_^a^	5.48 ± 0.49	4.76 ± 0.17	1.44 ± 0.09	1.03 ± 0.32	1.41 ± 0.33	1.91 ± 0.74	<0.001
WC^v^(mg/cm^3^)	1027.17 ± 4.42	1036.30 ± 1.68	1037.31 ± 4.72	1028.74 ± 9.55	1037.35 ± 6.48	1034.34 ± 5.93	0.054
IC^v^(×10^2^ μg/cm^3^)	36.19 ± 2.30	25.28 ± 0.46	9.35 ± 0.58	8.18 ± 1.10	10.88 ± 3.21	13.72 ± 3.44	<0.001
NIC^v^	0.597 ± 0.038	0.410 ± 0.008	0.248 ± 0.029	0.190 ± 0.029	0.280 ± 0.054	0.368 ± 0.170	<0.001
λ_HU_^v^	5.41 ± 0.56	3.82 ± 0.07	1.48 ± 0.11	1.27 ± 0.17	1.69 ± 0.46	2.18 ± 0.54	<0.001

BF, blood flow; BV, blood volume; MTT, mean transit time; PS, permeability surface.

WC, water concentration; IC iodine concentration; NIC, normalized iodine concentration; λ_HU_, slope of spectral HU curve.

a, the optimal arterial phase; v, the optimal venous phase.

TC, thymic carcinoma.

For the perfusion parameters, the BF and BV values of Type A and AB were significantly higher than those of Type B3 and TC (*P* < 0.05). The PS values of Type AB were higher than those of Type B1 (*P* = 0.030).

For the spectral parameters, IC^a^, NIC^a^, λ_HU_^a^, IC^v^, NIC^v^ and λ_HU_^v^ values of Type A or AB were significantly higher than those of Types B1, B2 and B3 (all *P* < 0.05), while there was no significant difference among Types B1, B2, B3 and TC or between Types A and AB (all *P* > 0.05).

### DECT perfusion imaging parameters of the two simplified groups of 51 TETs

The relationship between the perfusion and spectral parameters and each of the two simplified groups is shown in Table 5. For the perfusion and spectral parameters, the differences in MTT, PS, WC^a^ and WC^v^ among the three subgroups within Simplified Group 1 were not statistically significant (all *P* > 0.05). The opposite was found for the other parameters. PS in Simplified Group 2 was statistically significant, and the other parameters showed trends similar to those in Simplified Group 1. The IC^v^, NIC^v^ and λ_HU_^v^ values for HRT* were lower than those for TC (all *P* < 0.05).

**Table 5:** Perfusion and spectral parameters in Simplified Groups 1 and 2 of 51 TETs.

Perfusion, Spectral parameters	Simplified Group 1			*P*	Simplified Group 2			*P*
	LRT (A/AB/B1) (n = 16)	HRT (B2/B3) (n = 18)	TC (n = 17)		LRT* (A/AB) (n = 9)	HRT* (B1/B2/B3) (n = 25)	TC (n = 17)	
BF(ml/min/100 g)	113.20 ± 53.77	59.91 ± 20.68	53.62 ± 13.84	0.004	156.17 ± 19.05	59.36 ± 20.17	53.62 ± 13.84	<0.001
BV(ml/100 g)	14.59 ± 10.10	5.96 ± 1.59	6.21 ± 1.89	0.006	20.70 ± 9.41	6.18 ± 2.05	6.21 ± 1.89	<0.001
MTT(s)	9.37 ± 2.43	8.43 ± 2.30	9.86 ± 4.07	0.370	9.73 ± 3.04	8.56 ± 2.07	9.86 ± 4.07	0.515
PS(ml/min/100 g)	26.78 ± 13.18	22.44 ± 9.26	28.92 ± 11.68	0.264	35.83 ± 8.41	20.40 ± 9.31	28.92 ± 11.68	0.001
WC^a^(mg/cm^3^)	1037.53 ± 3.85	1033.76 ± 4.98	1034.78 ± 5.55	0.108	1035.47 ± 3.93	1035.55 ± 5.16	1034.78 ± 5.55	0.878
IC^a^ (×10^2^ μg/cm^3^)	22.74 ± 13.22	7.60 ± 2.49	12.35 ± 4.94	<0.001	33.41 ± 5.88	7.99 ± 2.21	12.35 ± 4.94	<0.001
NIC^a^	0.146 ± 0.069	0.061 ± 0.017	0.090 ± 0.029	<0.001	0.196 ± 0.051	0.067 ± 0.017	0.090 ± 0.029	<0.001
λ_HU_^a^	3.49 ± 1.90	1.20 ± 0.37	1.91 ± 0.74	<0.001	5.08 ± 0.50	1.26 ± 0.33	1.91 ± 0.74	<0.001
WC^v^(mg/cm^3^)	1034.46 ± 5.71	1032.56 ± 9.21	1034.34 ± 5.93	0.859	1032.24 ± 5.65	1033.89 ± 8.39	1034.34 ± 5.93	0.598
IC^v^(×10^2^ μg/cm^3^)	21.04 ± 11.50	9.38 ± 2.60	13.72 ± 3.44	<0.001	30.13 ± 5.93	9.37 ± 2.21	13.72 ± 3.44	<0.001
NIC^v^	0.386 ± 0.147	0.230 ± 0.061	0.368 ± 0.170	0.001	0.493 ± 0.101	0.235 ± 0.054	0.368 ± 0.170	<0.001
λ_HU_^v^	3.19 ± 1.69	1.46 ± 0.39	2.18 ± 0.54	<0.001	4.52 ± 0.91	1.47 ± 0.33	2.18 ± 0.54	<0.001

BF, blood flow; BV, blood volume; MTT, mean transit time; PS, permeability surface.

WC, water concentration IC iodine concentration; NIC normalized iodine concentration; λ_HU,_ slope of spectral HU curve.

a, the optimal arterial phase; v, the optimal venous phase.

TC, thymic carcinoma.

### Receiver operating characteristic (ROC) curve results of PS, IC^v^, NIC^v^, and λ_HU_^v^ values differentiating HRT* from TC

**Figure 4:**
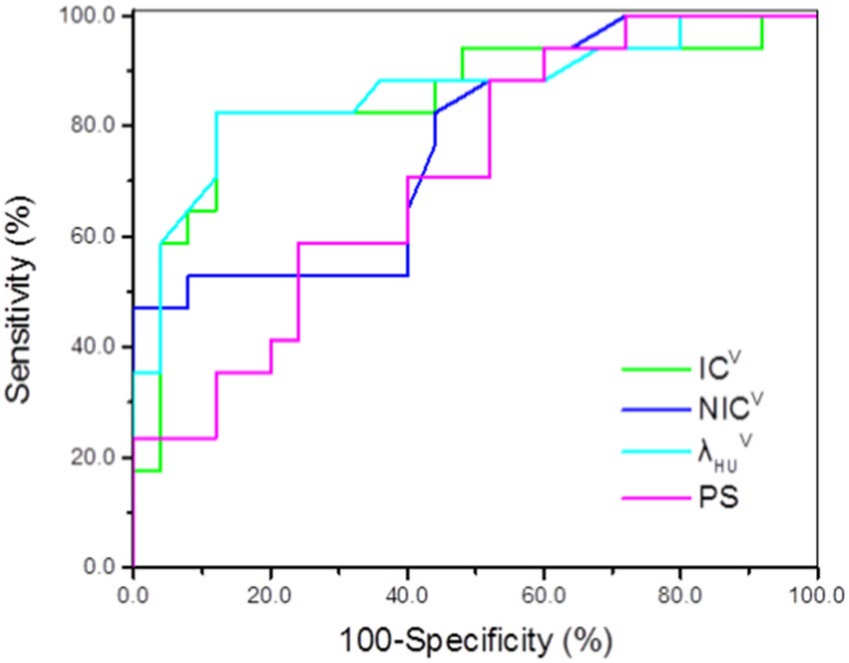
ROC result of PS, IC^v^, NIC^v^ and λ_HU_^v^ values differentiating HRT* from TC. The cutoff values of PS, IC^v^, NIC^v^, and λ_HU_^v^ values used for differentiating HRT* from TC were 17.40 mL/min/100 g, 11.42 × 10^2^ μg/cm^3^, 0.356, and 1.81, respectively, with AUC of 0.715,0.849, 0.769, and 0.862, respectively, sensitivity of 88.2%, 82.4%, 47.1%, and 82.4%, respectively, and specificity of 48.0%, 88.0%, 100.0%, and 88.0%, respectively, accuracy of 64.3%, 85.7%, 78.6%, 85.7%, respectively, PPV of 53.6%, 82.4%, 100.0%, 82.4%, respectively, NPV of 92.9%, 95.7%, 73.5%, 95.7%, respectively.

The cutoff values of PS, IC^v^, NIC^v^ and λ_HU_^v^ used to differentiate HRT* (Types B1, B2, B3) from TC in Simplified Group 2 were 17.40 ml/min/100 g, 11.42 × 10^2^ μg/cm^3^, 0.356, and 1.81, respectively (AUC: 0.715, 0.849, 0.769 and 0.862; sensitivity, 88.2%, 82.4%, 47.1% and 82.4%; specificity, 48.0%, 88.0%, 100.0%, and 88.0%; accuracy, 64.3%, 85.7%, 78.6%, 85.7%; PPV, 53.6%, 82.4%, 100.0%, 82.4%; NPV, 92.9%, 95.7%, 73.5%, 95.7%; [Fig. 4, Table 6]).

**Table 6:** ROC results of PS, IC^v^, NIC^v^, and λ_HU_^v^ values differentiating HRT* from TC.

HRT* vs. TC	*P*	cutoff value	AUC	Sensitivity (%)	Specificity (%)	Accuracy(%)	PPV(%)	NPV(%)
PS	0.019	<17.40 ml/min/100 g	0.715	88.2	48.0	64.3	53.6	92.9
IC^v^	0.005	<11.42 × 10^2^ μg/cm^3^	0.849	82.4	88.0	85.7	82.4	95.7
NIC^v^	0.010	<0.356	0.769	47.1	100.0	78.6	100.0	73.5
λ_HU_^v^	0.003	<1.81	0.862	82.4	88.0	85.7	82.4	95.7

ROC, receiver operating characteristic curve; AUC, area under the receiver operating characteristic curve.

PS, permeability surface; IC, iodine concentration; NIC, normalized iodine concentration; λ_HU_, slope of spectral HU curve; V, the optimal venous phase.

NPV, negative predictive value; PPV, positive predictive value.

TC, thymic carcinoma.

## Discussion

The present study showed that the quantitative and semiquantitative parameters of conventional CECT imaging and DECT perfusion imaging could help to distinguish different pathological types and risk subgroups of TETs. DECT perfusion imaging could differentiate type B thymomas from TCs, while CECT imaging could not. In addition, partial conventional CT features, such as tumour size, calcification, MNFS and CT stage, were included.

For conventional CECT imaging, the mean CEmax, the only conventional CT quantitative parameter in this study, of Type A and AB were higher than those of the other types. Pan *et al*.^25^ found that the short-spindled pattern of Type A and AB may commonly arrange in a haemangiopericytic or microcystic pattern, which may explain why a higher degree of CT enhancement was observed in the above two subtypes in our study. This is consistent with the results of Hu *et al*.^19^, who first reported that the degree of CT enhancement and MNFS could preoperatively help determine the WHO pathological subtypes of TET patients, especially for the low-risk (type A and AB) and high-risk (types B1 B2, B3 and thymic carcinoma) subtypes. In this study, we found that MNFS was significantly different among the six pathological subtypes of TETs. However, we also found that the mean CEmax was not significantly different among types B1, B2, B3 and TC, as well as between HRT (Types B2, B3) and TC and between HRT* (Types B1, B2, B3) and TC (all *P* > 0.05). Hu *et al*. reported that a greater average tumour diameter indicated a higher probability that the tumour was malignant. Approximately 49.1% of the tumours were thymic cancers larger than 8 cm, while in our study, this percentage was approximately 77.4% (24/31).

The perfusion parameters reflect tissue vascularization and angiogenesis^26^. In theory, tumours from different tissues or different pathological types of the same tumour can be identified using these parameters. CT perfusion imaging has been used in the differential diagnosis of squamous cell carcinoma of the head and neck, as well as in the prognosis and posttreatment response evaluation of neck lymphoma^27–29^. Each pure substance has a specific spectral attenuation curve, and X-ray attenuation in various tissues can be expressed by a pair of known pure substances^30,31^. Based on these principles, spectral CT imaging could provide semiquantitative and quantitative parameters from the ROIs of tumours.

DECT perfusion imaging can provide perfusion and spectral parameters from tumours simultaneously through a one-stop CT perfusion scan in GSI mode. In the recent literature, there is no study concerning the differences in perfusion or/and spectral parameters among the six WHO pathological subtypes, whereas a large number of studies comparing LRT (Types A, AB and B1), HRT (Types B2, B3) and TC have been conducted^16–23^. In our study, quantitative analyses of the majority of perfusion and spectral parameter values revealed differences between the different WHO pathological subtypes, especially between Type A or AB and Type B (B1, B2, B3) or TC. However, there was no significant difference among Types B1, B2, B3, and TC; this result was the same as for mean CEmax. We noted a certain overlap among some of the six subtypes, which made it difficult to distinguish them. Although Type B1 belongs to the low-risk group, its mean CEmax and spectral and perfusion parameter values are similar to those of Type B2 and B3. Pathologically, type B thymomas apparently represent a continuum from B1 to B3 thymomas that show a spectrum of lymphocyte to epithelial predominance^32^. Therefore, pathologists may experience an overlap in the diagnosis of type B1 and B2 thymomas (approximately 15% disagreement)^4^.

Based on the above results, we hypothesized that regrouping would be more conducive for the identification of TETs by radiologists. Thus, we regrouped the parameters into three new subgroups (LRT*: Types A, AB, HRT*: Types B1, B2 and B3; TC) and compared them. After the analyses, we concluded that there were statistically significant differences among the three new subgroups, especially between HRT* and TC based on the PS, IC^v^, NIC^v^, and λ_HU_^v^ values. The four parameter values were lower in the high-risk TET* (Types B1, B2, and B3) group than for thymic carcinoma, and the cutoff values used to differentiate them were 17.40 ml/min/100 g, 11.42 × 10^2^ μg/cm^3^, 0.356, and 1.81, respectively. These four values demonstrated large AUCs and high sensitivities and specificities for differential diagnosis (Table 6).

PS is a diffusion coefficient that reflects the one-way transmission speed of contrast agents through the capillary endothelium into intercellular space. It is strongly correlated with the integrity of endothelial cells and the inter-cellular space of the tissues^33^. The new vessel wall in HRT* is more mature than that in TC, and the permeability of the vessel wall is lower. Therefore, this provides an explanation for the lower PS level in HRT* than in TC in our study. Compared with PS, the three spectral parameters have greater AUC, specificity and accuracy values, which may mean that the spectral parameters of the optimal venous phase have greater value in identifying HRT* and TC than the perfusion parameters. Some scholars have made similar findings on the CT perfusion parameters and IC value in differentiating cervical Hodgkin’s lymphoma from non-Hodgkin’s lymphoma, and they have suggested that iodine concentration could better reflect blood perfusion in tumours^13^. The application of NIC helps to prevent some errors due to various medical reasons, such as differences in patient condition, blood vessel properties and cardiac function. The specificity of NIC^v^ values in differentiating HRT* from TC was 100% and was higher than that (88.0%) of IC^v^. Coursey^34^ *et al*. found that the CT value changed greatly in the low energy range of the spectral curve. Therefore, a low energy range (40–80 keV) should be selected to analyse the images to display the difference in the slopes (λ_HU_) adequately. In particular, there were some significant differences among the three subgroups (LRT*, HRT*, TC) with respect to the differential diagnoses. Yan^35^ *et al*. found that the IC value in the venous phase yielded the highest performance for differentiating LRT from HRT or TC, which was consistent with our results. However, they found no significant difference between HRT and TC, which was the opposite of our results. This may be because the principle of single- and dual-source CT is different. Therefore, it is necessary to carry out this study with two different kinds of CT scanner. In addition, there was no significant difference between HRT* and TC in any of the spectral parameters of the optimal arterial phase. The high concentration of contrast agent in the superior vena cava or right atrium in the optimal arterial phase results in a more obvious partial volume effect in the adjacent lesion area than in the optimal venous phase, so the authenticity of ROI data may be affected. We speculated that this was one of the reasons for this phenomenon.

We would like to specify the selection principles for the ROIs. In this study, we found that the standard deviation of some of the perfusion parameter values of small freehand ROIs (<200 mm^2^) measured on the perfusion pseudocolour image was relatively large. Therefore, we aimed to choose relatively large ROIs (200–1000 mm^2^) while avoiding selecting haemorrhagic, necrotic, cystic and calcification areas in the tumour to minimize measurement errors. However, no such phenomenon was found in the single-energy images.

This study had some limitations. First, only a small number of patients had TETs, which limited the statistical power of the study. Rare types of thymoma, such as micronodular thymoma and atypical Type A variant thymoma, were not included in the study. Additional studies with a larger number of patients will be needed to verify our results. Second, the length of some tumours exceeded the DECT perfusion scan range, which was limited to the 40 mm z-axis coverage. Third, the grey level of the anterior mediastinal mass is susceptible to a partial volume effect on CT images, especially for smaller lesions, although we excluded lesions less than 2.0 cm in diameter. Fourth, all semiquantitative and quantitative parameter values were measured three times by a radiologist, and there was no assessment of the repeatability and consistency of the data. In addition, we used a relatively high radiation dose in the perfusion region (40 mm z-axis coverage).

In conclusion, the parameters of conventional CECT imaging and DECT perfusion imaging have important diagnostic value in identifying different pathologic subtypes of TETs, especially those of DECT perfusion imaging in HRT* (Types B1, B2, B3) and thymic carcinoma.

## Methods

### Patients

This study was approved by the Ethics Committee of Shanxi Cancer Hospital, and written informed consent was obtained from all patients. This study included 123 adult patients with untreated anterior mediastinal lesions suspected of having thymic tumours. These lesions were detected using CT imaging from June 2014 to September 2017. This study was conducted in accordance with the Declaration of Helsinki. Before the scan, the physicians in charge confirmed whether the patient had received a conventional CT enhancement scan or a DECT perfusion scan, and the patients and their family members were required to sign a confirmation form. Written informed consent from the patients and institutional review board approval were obtained. The inclusion criteria were as follows: (a) solid anterior mediastinal TETs; (b) lesions> 2.0 cm in diameter based on the longest diameter; and (c) patients who had not undergone biopsy, treatment with chemotherapy, radiation therapy, or surgery before the CT scan. The exclusion criteria were patients with mediastinal TETs without specific pathological subtypes (n = 4), lymphoma (n = 14), neuroendocrine tumours (n = 7), thymic cysts (n = 5), thymic hyperplasia (n = 2), bronchogenic cysts (n = 1), and poor quality perfusion CT images caused by motion artefacts (n = 2). Finally, 88 patients (mean age: 54.02 ± 9.93 years, age range: 31–78 years) with TETs were enrolled: 42 men (mean age: 58.74 ± 9.76 years, age range: 43–77 years) and 46 women (mean age: 57.18 ± 8.72 years, age range: 31–78 years). Furthermore, 30 patients were asymptomatic; their tumours were detected after chest radiography or CT. In the symptomatic patients (58 patients), the patients presented with chest pain or discomfort (n = 27), symptoms and signs of myasthenia gravis (n = 17), respiratory symptoms (n = 15), and others (n = 7). The final diagnosis was based on surgery (n = 66) and percutaneous biopsy (n = 22) with histopathologic examination.

### Conventional CECT scan

Thirty-seven patients with TETs underwent conventional CECT scan (the routine scanning sequence for chest tumours) using a GE Discovery CT 750HD. Before scanning, patients were instructed to hold their breath to avoid motion artefacts. The chest scan protocol (manufacturer number-5.3 Three phase chest c−/c + ) was used. The scan range was from the thoracic inlet to the diaphragmatic level. The first series was a thorax precontrast CT study (helical scan type, 100 kV and automatic mAs, rotation time 0.6 s, slice reconstruction and interval 5 mm each, pitch 1.375:1). A total of 40 to 120 mL (1 mL/kg weight) of contrast medium (iohexol, 300 mg/mL, iodine) was injected by using a pump injector at a rate of 3.0 mL/s. Arterial phase scanning began 11 s after the trigger attenuation threshold (120 HU) reached the level of the thoracic aorta. Venous phase scanning began at a delay of 25 s after arterial phase scanning. Scanning parameters were the same as in the plain CT. All imaging data underwent further multiplanar reconstruction and were analysed at a medical imaging workstation.

### DECT perfusion scan

Fifty-one patients with TETs underwent DECT perfusion imaging using the GE Discovery CT 750HD. Before the CT scanning, patients were instructed to hold their breath to avoid motion artefacts. The first series, a thorax precontrast CT study, was performed to identify the tumour as in the conventional CT enhancement scan. The second series was a chest gemstone spectral imaging (GSI) mode scan protocol (manufacturer number-5.27 Perfusion GSI) study (tube voltage fast switching between 80 kVp and 140 kVp), for which 1.0 ml/kg of the non-ionic iodinated contrast (iohexol, 300 mg/ml iodine), followed by 30 ml of saline, was administered intravenously using the pump injector (Stellant, United States) at a flow rate of 5 ml/s on the median cubital vein. Eight contagious 5-mm-thick reconstructed sections (total z-axis coverage of 40 mm), which were previously chosen in the precontrast series, were obtained. Image acquisition started in axial (continuous) mode after 6 s of contrast injection. A total of 25 cycles were run while maintaining an intercycle interval of 2 seconds. The total scan duration was 50 s (200 images per study). Patients were advised to breathe quietly, stay motionless, and not swallow during the dynamic CT scanning. The third series was performed in the same way as the first series. The scan ranges of the first and third series were all from the thoracic inlet to the diaphragmatic level. After scanning, the images were transferred to an AW4.5 workstation.

### CT image processing and data acquisition

The conventional CECT scan series were analysed at the workstation. The maximal difference in CT values between the precontrast scan and the contrast-enhanced phases in the solid component of the tumour was denoted as the maximal contrast-enhanced range (CEmax). The region of interests (ROIs) were manually placed so that they were smaller in size than the mass by avoiding bias from small regions of necrosis, cystic elements or calcification. The final CEmax was the average of the maximal difference values of the ROIs of the three selected sections.

The DECT perfusion scan series were analysed with the Perfusion 4 software package. Arterial input was defined by the 40 to 100 mm^2^ circular ROI that was placed in the aortic arch or thoracic aorta at the site of the largest level of the tumour. Freehand ROIs (200–1000 mm^2^ quasi-circular area) were drawn around the TETs, taking care to avoid areas of cystic necrosis, calcifications or tumour blood vessels. The arterial time-density curve (TDC) was derived automatically, and parametric (BF, BV, MTT, PS) coloured maps were displayed for each of the eight contagious series of the perfusion CT. The optimal arterial and venous phases were determined by the numbers of scanning periods corresponding to the first and second peaks on the TDC curve (Fig. 3b). Thereafter, the mixed energy images were reconstructed to single-energy images of approximately 70 keV with the material decomposition (MD) analysis software package. Single energy maps of the optimal arterial phase and venous phase were obtained with 50% adaptive statistical iterative reconstruction (ASIR) to reduce noise and improve image resolution. Freehand ROIs (40–100 mm^2^ circular area) were drawn in the solid areas of the tumours with the GSI viewer. We used the copy and paste function in the workstation to ensure consistent ROIs for the same patient in the optimal arterial and venous phases. Moreover, we obtained WC and IC through the measurement of iodine-based and water-based images. Normalized iodine concentrations (NICs) and λ_HU_ were calculated separately using the following formula:

NIC = IC_tumour_/IC_Thoracic aorta or aortic arch;_ λ_HU_ = (CT_40keV_-CT_80keV_)/40. All semi-quantitative and quantitative parameter values were measured by one radiologist (with over 12 years of experience in radiology) at different levels of the tumour three times and then averaged.

The partial conventional CT features of 88 TETs were evaluated according to the following rules: (1) Size: the longest diameter of the tumour was measured where the tumour appeared largest on an axial enhanced CT image; (2) Multiple nodules with fibrous septa (MNFS): multiple enhancement nodules with different sizes and linear low-density structures between them on enhanced CT images.

### Statistical analysis

Numerical variables are reported as the means and standard deviations. Between-group comparisons for gender, Masaoka-Koga stage and partial conventional CT features (including tumour size, calcification and MNFS) were conducted using the chi-squared (*χ*^2^) test. Between-group comparisons for age, mean CEmax values and perfusion and spectral parameter values were conducted using the Kruskal-Wallis test. Receiver operating characteristic (ROC) curve analyses were performed to determine the optimum cutoff for differentiating HRT* (Types B1, B2, and B3) from TC in Simplified Group 2 using perfusion and spectral parameters and calculate the sensitivity, specificity, and area under the ROC curve (AUC) at one time. *P* < 0.05 indicated a statistically significant difference. SPSS 22.0 software was used for statistical analyses.
