# Author Correction: Dual-energy CT perfusion imaging for differentiating WHO subtypes of thymic epithelial tumors

**DOI:** 10.1038/s41598-020-64341-5

**Published:** 2020-04-29

**Authors:** Chunhai Yu, Ting Li, Ruiping Zhang, Xiaotang Yang, Zhao Yang, Lei Xin, Zhikai Zhao

**Affiliations:** 1 Imaging Department, Shanxi Tumor Hospital, The Affiliated Tumor Hospital of Shanxi Medical University, Taiyuan, Shanxi 030013 P.R. China; 2 Department of Nephrology, Taiyuan People’s Hospital, Taiyuan, Shanxi 030001 P.R. China

Correction to: *Scientific Reports* 10.1038/s41598-020-62466-1, published online 26 March 2020

This Article contains an incorrect version of Figure 3. The correct Figure 3 has been reproduced below as Figure 1.

**Figure 1:**
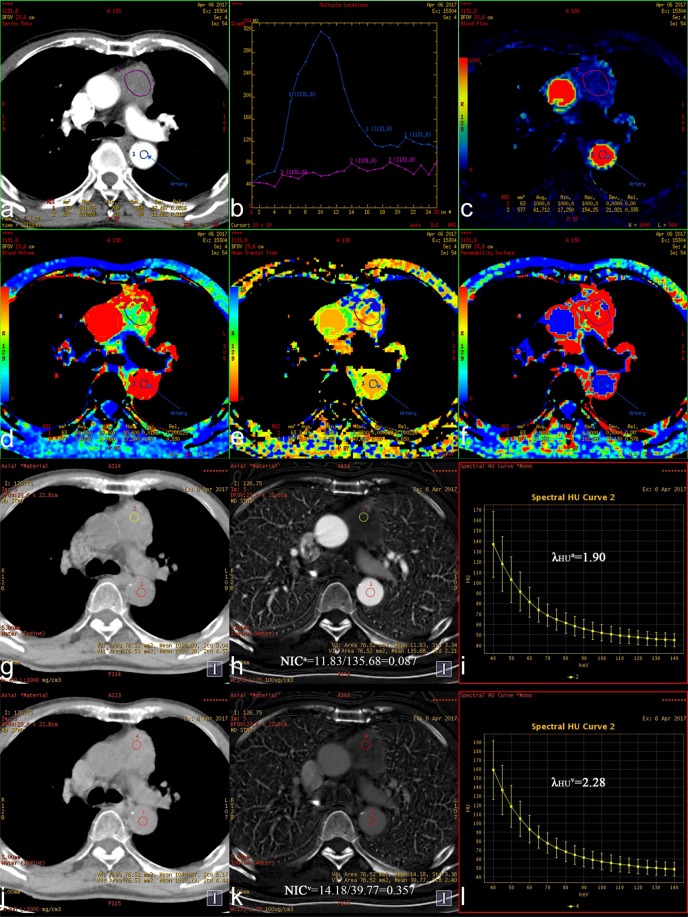
.

Additionally, there is an error in the Methods section.

“This study was approved by the Ethics Committee of Shanxi Cancer Hospital, and written informed consent was obtained from all patients.”

should read:

“This study was approved by the Ethics Committee of Shanxi Tumor Hospital, and written informed consent was obtained from all patients.”

